# Multiomics data integration unveils core transcriptional regulatory networks governing cell-type identity

**DOI:** 10.1038/s41540-020-00148-4

**Published:** 2020-08-24

**Authors:** Sascha Jung, Antonio del Sol

**Affiliations:** 1 CIC bioGUNE, Bizkaia Technology Park, 48160 Derio, Spain; 2 Computational Biology Group, Luxembourg Centre for Systems Biomedicine (LCSB), University of Luxembourg, 4362 Esch-sur-Alzette, Luxembourg; 3 IKERBASQUE, Basque Foundation for Science, 48013 Bilbao, Spain; 4 Moscow Institute of Physics and Technology, Dolgoprudny, 141701 Russia

**Keywords:** Software, Molecular biology

## Abstract

A plethora of computational approaches have been proposed for reconstructing gene regulatory networks (GRNs) from gene expression data. However, gene regulatory processes are often too complex to predict from the transcriptome alone. Here, we present a computational method, Moni, that systematically integrates epigenetics, transcriptomics, and protein–protein interactions to reconstruct GRNs among core transcription factors and their co-factors governing cell identity. We applied Moni to 57 datasets of human cell types and lines and demonstrate that it can accurately infer GRNs, thereby outperforming state-of-the-art methods.

## Introduction

Cellular phenotypes are characterized by stable gene expression profiles maintained by a set of transcription factors (TFs) that jointly determine cell identity. Together with other co-factors, these identity TFs form a regulatory core network, which is shaped by the epigenetic landscape^1–3^. In particular, the epigenetic landscape, defined by epigenetic modifications, chromatin accessibility, and chromatin conformation, determines phenotype-specific active regulatory regions of each core TF^4^. In particular, in contrast to active promoter regions shared by various cell and tissue types, enhancer regions modulate phenotype-specific expression profiles due to their phenotype-specific selection^5^.

In the past decades, a wealth of computational methods has been developed that aim at identifying regulatory interactions between genes^6–11^. However, these methods require tremendous amounts of transcriptomics data and cannot provide information about the active regulatory regions, an issue that even new technologies such as single-cell profiling do not mitigate.

To address these shortcomings, we propose Moni (*M*ulti-*o*mics *n*etwork *i*nference), a computational method that systematically integrates histone modification, chromatin accessibility, and transcriptomics data with a global atlas of TF-binding events, enhancer–promoter interactions, and protein–protein interactions across diverse cell types and lines, obtained from ENCODE^12^, the Roadmap Epigenomics Project^13^ and the Blueprint database^14^, in order to reconstruct phenotype-specific core regulatory networks. As a result, Moni provides a comprehensive map of the phenotype-specific core regulatory network including regulation at distal enhancer regions and the cooperativity of TFs in the regulation of target genes. With the steady increase in epigenetic profiling, we expect Moni to be of general utility for the molecular characterization of cellular phenotypes and to aid in the identification of key regulators.

## Results and discussion

**Fig. 1:**
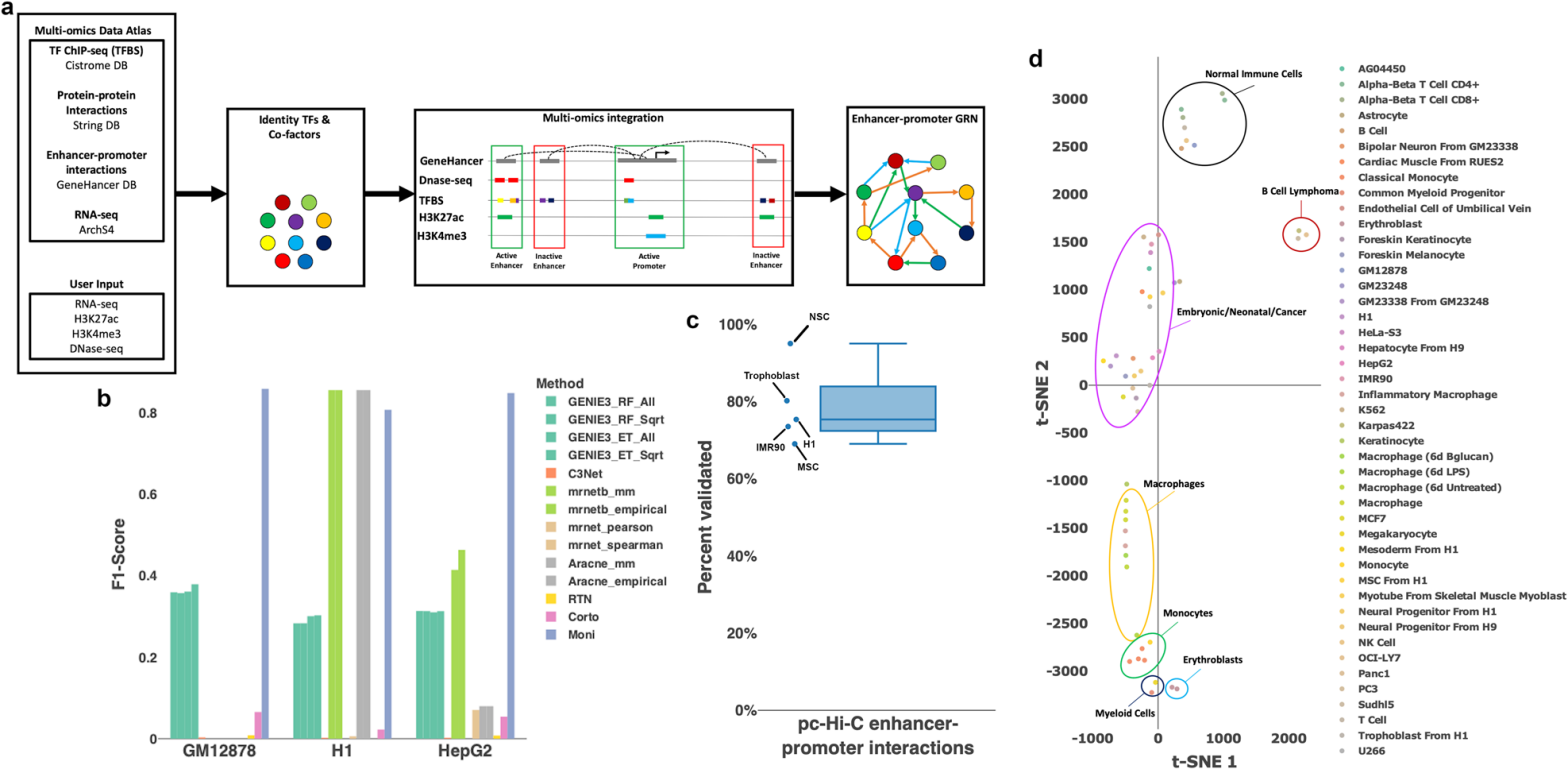
Method overview and benchmark results. **a** Overview of the methods workflow. **b** Benchmark results against state-of-the-art methods. Gold-standard networks for GM12878, H1, and HepG2 were from cell-type-specific TF ChIP-seq data and mapped to promoter regions. Each methods ability to identify these interactions is expressed as the F1 score. **c** Benchmark results of enhancer–promoter assignments of the proposed method. A gold-standard dataset of cell-type-specific promoter-capture Hi-C data was assembled H1 embryonic stem cells, IMR90 fibroblasts, mesenchymal stem cells (MSC), neural stem cells (NSC), and trophoblasts. The percentage of validated enhancer–promoter assignments is reported as a box plot showing the median (solid center line). Whiskers extend to 1.5 times the interquartile range. Between 69% (MSC) and 95% (NSC) of interactions could be validated. **d** t-SNE plot of cell types and cell lines reconstructed with Moni. Seven clusters were identified that are specific to different cell types.

Given a particular phenotype, gene regulatory network (GRN) reconstruction by Moni involves three main steps (Fig. 1a). Firstly, core TFs are detected by comparing the expression of each TF to their expression in a background datasets of other cell types and lines assembled from ArchS4 (ref. ^15^). Based on a previous study demonstrating that the TFs with the highest phenotypic specificity are most likely to be essential determinants of cell identity^16^, the 10 TFs with the highest phenotypic specificity are selected as core TFs. In addition, potential co-factors are detected, i.e., TFs that are significantly more specific to the phenotype than their expected median specificity. Secondly, active promoters and enhancers of core TFs and co-factors are identified. Promoter regions are considered to be active if they overlap with at least one H3K4me3 peak while potential enhancers are associated to TFs on the basis of the GeneHancer database^17^ and deemed active if they overlap with at least one H3K27ac peak. Finally, directed interactions among core TFs and co-factors are inferred if they satisfy each of the following conditions: (1) the promoter of the target TF is active, (2) the interaction is supported by a (non-phenotype-specific) ChIP-seq peak in the promoter or any active enhancer region of the target TF, and (3) the supporting ChIP-seq peak falls within an accessible chromatin region. For interactions within the same genomic regions, i.e., enhancer or promoter, cooperative and competitive TF regulation is determined by the overlap of supporting ChIP-seq peaks and whether a protein–protein interaction among the TFs has been reported in databases (Supplementary Fig. 1).

We first set out to validate the accuracy of the reconstructed regulatory interactions in three well-characterized cell lines, namely H1 (embryonic stem cells), GM12878 (B-lymphocytes), and HepG2 (liver cancer), and compared the performance to six state-of-the-art methods including GENIE3 (ref. ^6^), RTN^9,10^, ARACNE^18^, and Minet^8^. Cell-type-specific TF ChIP-seq data were collected from Cistrome^19^ and ENCODE^12^ and restricted to TFs in the networks reconstructed by each individual method. Comparison of predicted and validated interactions in the promoter regions resulted in an average F1 score of 0.84 of Moni (Fig. 1b). In contrast, the best performing state-of-the-art methods, ARACNE and Minet, only achieved average F1 scores between 0.31 and 0.44 (Fig. 1b).

Next, we validated the enhancer–promoter assignment by our method in five cell lines and types, namely H1, IMR90 (lung fibroblast), in vitro-differentiated mesenchymal and neural stem cells, and in vitro-differentiated trophoblast, using promoter-capture Hi-C datasets^20^. As a result, we were able to validate on average 78.6% of enhancer–promoter interactions with up to 95% validated interactions in neural stem cells (Fig. 1c). Moreover, the overall distribution of enhancers in reconstructed GRNs of 54 cell lines and types from ENCODE^12^, Roadmap Epigenomics^13^, and Blueprint^14^ follows an exponential distribution where most of the TFs have only one or two enhancers (Supplementary Fig. 2), which is consistent with previous experimental studies^21^.

In addition to validating interactions and enhancer–promoter assignments, we assessed the selection and reproducibility of core TFs by employing the reconstructed networks for 54 cell lines and types. Projecting the incidence matrix of TFs and cell types into planar space reveals a homogeneous clustering by cell types, except for embryonic and cancer samples that are clustered together due to their share of co-factors (Fig. 1d). Of note, the clustering of embryonic and cancer samples can be further divided into smaller sub-clusters of samples of the same cell type or lineage. For instance, the cancer cell lines MCF-7, PANC-1, and PC3 are closely related to neonatal foreskin keratinocytes. Indeed, all of these cells possess an epithelial phenotype, which is reflected in the TFs selected by Moni. Similarly, embryonic stem cell-derived hepatocytes and HepG2 cells form a smaller sub-cluster representing the hepatocyte identity while individual differences in the selected TFs establish the differences between cancer and normal samples. Moreover, hierarchical clustering of the incidence matrix supports these conclusions by consistently relating both broad cell types and subtypes, for example, induced by treatment with compounds (Supplementary Fig. 3). In particular, three clusters of TFs could be identified: (i) co-factors predominantly active in blood cells, (ii) co-factors predominantly active in non-blood cells, and (iii) phenotype-specific core TFs. Importantly, Moni identified RUNX1, a known pioneer factor, as a co-factor rather than a core TF due to its expression in multiple cell types, such as fibroblasts, erythroblasts, and monocytes (Supplementary Fig. 4). This finding supports Moni’s underlying hypothesis that a combination of core TFs and core factors are necessary to convey cell-type identity.

Next, we sought to establish the functional implication of core TFs and co-factors in maintaining important cell-type functions. Gene Ontology enrichment demonstrates that the selected TFs are not only consistently selected but are also implicated in important cellular functions (Supplementary Table 1). For example, induced pluripotent stem cells express well known pluripotency factors, i.e., NANOG, SOX2, and POU5F1, and are enriched in categories corresponding to stem cell maintenance (Supplementary Fig. 4). Moreover, CD4-positive alpha–beta T cells are enriched in “immune response”, “T cell activation” and, strikingly, “CD4-positive, alpha–beta T cell activation”. Thus, Moni is able to select core TFs and co-factors governing cell-type- and subtype-specific biological processes.

After establishing Moni’s accuracy in selecting TFs governing cell identity and its increased accuracy of reconstructed networks between these TFs, we set out to validate the predicted TF complexes jointly regulating their target genes. Due to the increased availability of previous studies, we focused our analysis on H1 embryonic stem cells. Moni identified in total 12 protein complexes involving between 2 and 4 TFs. Of these, ten complexes involve NANOG, SOX2, or POU5F1 and have been validated in previous studies or curated databases. In addition, the remaining two complexes, TCF12-TCF4 and TCF7L1-TCF7L2, have been previously verified, as well (Supplementary Table 2).

In summary, Moni integrates multiple-omics datasets to provide a more comprehensive characterization of the core GRN controlling cell identity. Although, we demonstrated the performance of Moni using human cell types and lines in this study, it is applicable to samples from other species. Especially for mouse cells, a multi-omics data atlas can be readily compiled from data in ArchS4 (ref. ^15^), Cistrome^19^, String^22^, and Enhancer Atlas^23^. In addition, publicly available query datasets are available from the International Human Epigenome Consortium^24^ (IHEC), ENCODE^12^, and Gene Expression Omnibus^25^. Thus, we expect Moni to be a valuable tool for obtaining mechanistic insights into key transcriptional regulators in processes such as cell conversion and disease phenotypes.

## Methods

### Assembly of background distribution

We collected all human RNA-seq count data from ArchS4 (version 8, 2/2020)^15^ and selected polyA and total RNA samples based on the provided metadata. Next, we removed single-cell experiments and samples with a low number of counts. Based on the distribution of counts of each sample, we removed samples with less than 15,000 counts. PolyA and total RNA-seq samples were divided into distinct sets for assembly of distinct background distributions. We further removed correlated samples in each set following two steps. First, select a random sample as the seed and, second, iteratively add randomly selected samples whose Pearson correlation coefficient to all already added samples is less than 0.7. After removal of correlated samples, we end up with 583 uncorrelated polyA RNA-seq samples and 2523 total RNA-seq samples serving as the background distributions for each gene. Finally, all counts were transformed to transcripts per million.

### Identification of identity TFs and co-factors

Given a query RNA-seq sample, the appropriate background distribution, i.e., polyA or total RNA, was selected based on the sequenced molecules. For each TF, the following three steps were performed. First, an idealized distribution was created for the query and background samples, having probability “1” in place of the query sample and “0” otherwise. Second, the background expression distribution was created for the combined query and background samples. Probabilities are defined as the scaled expression in each sample such that the sum of probabilities equals one. Finally, Jennsen–Shannon divergence (JSD) was computed between the idealized and background distributions. The 10 TFs having the lowest JSD value were considered identity TFs.

For co-factors, JSD values were computed for each sample in the background and ranked in ascending order, i.e the lowest JSD value has rank 1. Given a query sample, for each TF a *z*-score was computed for the rank its JSD value given the distribution of ranks in the background sample. Each TF having a *z*-score less than −1.5 were considered as co-factors.

### Reconstruction of core GRNs

GRN reconstruction follows a four-step approach. First, identity TFs and co-factors are selected as described before. Second, active proximal and distal regulatory regions are identified for every identity TF and co-factor. Promoters are defined on the basis of the Ensembl promoter annotation from the Eukaryotic Promoter Database^26^ and truncated to 1500 bp up- and 500 bp downstream. If a promoter overlaps at least one phenotype-specific H3K4me3 peak, it is considered to be active. In addition, potential enhancer regions are linked to active promoters by the Genehancer database^17^. Enhancers are active if they overlap at least one phenotype-specific H3K27ac peak and truncated to the peak regions. Inactive enhancers are not considered. Third, phenotype-specific DNase-seq peaks in active promoter and enhancer regions define potentially active regulatory sites. Finally, (non-specific) TF ChIP-seq peaks from Cistrome^19^ overlapping with an active regulatory site establishes the GRN scaffold. The GRN scaffold is further filtered such that only interactions between identity TFs and co-factors are retained that satisfy the following condition. Each co-factor included in the network must regulate at least one identity TF and must be regulated by at least one identity TF.

### Distinguishing cooperative and competitive TF binding

Cooperative and competitive binding events of overlapping TF ChIP-seq peaks is distinguished on the basis of two criteria. First, a protein–protein interaction is reported in the String database^22^ with a confidence score greater than 800. Second, the TF ChIP-seq peaks reciprocally overlap by at least 62%. The overlap threshold was defined on the basis of a positive and negative gold-standard dataset including 755 and 336 protein-protein interactions (PPIs), respectively^27,28^. The overlap of ChIP-seq peaks of interacting and non-interacting TFs was computed in all cell lines and types with available information in Cistrome^19^, resulting in a threshold of 62.43% above which TFs are more likely to interact than not (Supplementary Fig. 1). Multiple TFs fulfilling this condition in the same regulatory region are detected by constructing an undirected graph where edges represent an overlap of more than 62%. The connected components in this graph are detected using the clusters-method of the R “igraph” library (version 1.2.4.2) and are deemed cooperative, i.e., forming a complex for regulation. All other TFs with overlapping ChIP-seq peaks are deemed competitive.

### Comparison to other methods

We compared the performance of our method to six other methods, namely GENIE3 (ref. ^6^), C3Net^7^, Minet^8^, ARACNE^18^, RTN^9,10^, and CorTo^11^. Of note, ARACNE was run using the implementation in the Minet R-package. For GENIE3 and Minet, different algorithms were employed for reconstructing networks. In particular, we employed GENIE3 using either the random forests or extra trees algorithm with all TFs (parameter: “ALL”) or a random selection of approximately 41 TFs (parameter: “SQRT”). For comparison, interactions within the top quartile were considered. Further, we employed Minet using the following configurations:

1. Method parameter: mrnet and estimator parameter either “spearman” or “pearson”.
2. Method parameter: aracne and estimator parameter either “mi.mm” or “mi.empirical” using “globalequalwidth” discretization.
3. Method parameter: mrnetb and estimator parameter either “mi.mm” or “mi.empirical” using “globalequalwidth” discretization.

Finally, CorTo requires the user to define a set of centroids, i.e., TFs acting as regulators of other TFs, which we defined to be the identity TFs and co-factors identified by our method.

Each method was run on three datasets of H1 cells, GM12878 cells, and HepG2 cells for which we collected 7, 18, and 10 homogeneously processed total RNA-seq datasets from ENCODE^12^. Since, except RTN, all methods do not provide information about the directionality of the interaction, both potential directionalities were considered for the comparison.

Cell-line-specific gold-standard TF ChIP-seq datasets were collected from Cistrome^19^ and ENCODE^12^ and restricted to peaks falling within promoter regions of TFs. To obtain a fair comparison, promoter regions are defined as described above, i.e., 1500 bp up- and 500 bp downstream of the TSS.

### Gold-standard promoter-capture Hi-C datasets

Processed promoter-capture Hi-C datasets for H1, IMR90 (lung fibroblast), in vitro-differentiated mesenchymal and neural stem cells and in vitro-differentiated trophoblast were obtained from a previous study^20^. Due to the heterogeneity observed in tissue samples from different donors, we restricted the gold-standard dataset to cell lines and in vitro-differentiated cells under controlled conditions.

For each sample, we obtained promoter–promoter and promoter–other interactions and identified all interactions where one genomic regions is located in an enhancer described in GeneHancer^17^ and the other region is located within the promoter of a TF using bedtools version 1.2.4.2 (ref. ^29^) and a custom R script.

We restricted the gold-standard datasets of every cell line/type to the TFs in the reconstructed networks and computed the percentage of correctly assigned enhancer regions.

### Reporting summary

Further information on research design is available in the Nature Research Reporting Summary linked to this article.

## Supplementary information

Reporting Summary

Supplementary Figures

Supplementary Table legends

Supplementary Table 1

Supplementary Table 2

Supplementary Table 3

## Data Availability

The datasets RNA-seq, DNase-seq, and histone modification ChIP-seq data analyzed during the current study are available in ENCODE (https://www.encodeproject.org), ArchS4 (https://amp.pharm.mssm.edu/archs4/), Blueprint Epigenomics (http://dcc.blueprint-epigenome.eu/#/experiments), and Gene Expression Omnibus (https://www.ncbi.nlm.nih.gov/geo/). The identifier of each dataset can be found in Supplementary Table 3. The TF ChIP-seq data that support the findings of this study are available from Cistrome but restrictions apply to the availability of these data, which were used under license for the current study, and so are not publicly available. Data are however available from the authors upon reasonable request and with permission of Cistrome.

## Code availability

The source code of Moni together with its documentation is available online at https://github.com/saschajung/Moni.
