# Supplementary material for: Multiomics data integration unveils core transcriptional regulatory networks governing cell-type identity
Source: NPJ Syst Biol Appl. 2020 Aug 24;6:26. doi: 10.1038/s41540-020-00148-4 (PMC7445234; doi:10.1038/s41540-020-00148-4)
Supplement: Supplementary file 1 — Reporting Summary [file 41540_2020_148_MOESM1_ESM.pdf]

nature research

Corresponding author(s): Antonio del Sol
Last updated by author(s): Jul 2, 2020

Reporting Summary

Nature Research wishes to improve the reproducibility of the work that we publish. This form provides structure for consistency and transparency in reporting. For further information on Nature Research policies, see our Editorial Policies and the Editorial Policy Checklist.

Statistics

For all statistical analyses, confirm that the following items are present in the figure legend, table legend, main text, or Methods section.

n/a	Confirmed	
☐	☒	The exact sample size (*n*) for each experimental group/condition, given as a discrete number and unit of measurement
☐	☒	A statement on whether measurements were taken from distinct samples or whether the same sample was measured repeatedly
☒	☐	The statistical test(s) used AND whether they are one- or two-sided<br>*Only common tests should be described solely by name; describe more complex techniques in the Methods section.*
☒	☐	A description of all covariates tested
☒	☐	A description of any assumptions or corrections, such as tests of normality and adjustment for multiple comparisons
☐	☒	A full description of the statistical parameters including central tendency (e.g. means) or other basic estimates (e.g. regression coefficient) AND variation (e.g. standard deviation) or associated estimates of uncertainty (e.g. confidence intervals)
☒	☐	For null hypothesis testing, the test statistic (e.g. *F*, *t*, *r*) with confidence intervals, effect sizes, degrees of freedom and *P* value noted<br>*Give P values as exact values whenever suitable.*
☒	☐	For Bayesian analysis, information on the choice of priors and Markov chain Monte Carlo settings
☒	☐	For hierarchical and complex designs, identification of the appropriate level for tests and full reporting of outcomes
☒	☐	Estimates of effect sizes (e.g. Cohen's *d*, Pearson's *r*), indicating how they were calculated

Our web collection on statistics for biologists contains articles on many of the points above.

Software and code

Policy information about availability of computer code

Data collection	No software was used to collect data.
Data analysis	Moni requires bedtools v2.24.2.1, R version 3.6 or greater and a UNIX environment to run shell scripts ('awk' required). Custom R scripts have been used to generate figures using the R-packages heatmaply (version 1.1.0) and plotly (version 4.9.2.1).

For manuscripts utilizing custom algorithms or software that are central to the research but not yet described in published literature, software must be made available to editors and reviewers. We strongly encourage code deposition in a community repository (e.g. GitHub). See the Nature Research guidelines for submitting code & software for further information.

Data

Policy information about availability of data

All manuscripts must include a data availability statement. This statement should provide the following information, where applicable:

- Accession codes, unique identifiers, or web links for publicly available datasets
- A list of figures that have associated raw data
- A description of any restrictions on data availability

The source code of Moni together with its documentation is available online at https://github.com/saschajung/Moni. The datasets RNA-seq, DNase-seq and histone modification ChIP-seq data analyzed during the current study are available in ENCODE (https://www.encodeproject.org), ArchS4 (https://amp.pharm.mssm.edu/archs4/), Blueprint Epigenomics (http://dcc.blueprint-epigenome.eu/#/experiments) and Gene Expression Omnibus (https://www.ncbi.nlm.nih.gov/geo/). The identifier of each dataset can be found in Supplementary Table 3. The TF ChIP-seq data that support the findings of this study are available from Cistrome but restrictions apply to the availability of these data, which were used under license for the current study, and so are not publicly available. Data are however available from the authors upon reasonable request and with permission of Cistrome.

Field-specific reporting

Please select the one below that is the best fit for your research. If you are not sure, read the appropriate sections before making your selection.

☒ Life sciences ☐ Behavioural & social sciences ☐ Ecological, evolutionary & environmental sciences

For a reference copy of the document with all sections, see nature.com/documents/nr-reporting-summary-flat.pdf

Life sciences study design

All studies must disclose on these points even when the disclosure is negative.

Sample size	No sample size calculation was performed. For the compiled global multi-omics atlas all available datasets in the related databases were considered. However, correlated RNA-seq samples were excluded from the background distribution computation to avoid cell type biases. For the query dataset, a single sample of each required type, i.e. RNA-seq, H3K4me3 ChIP-seq, H3K27ac ChIP-seq and DNase-seq, is sufficient for generating the core networks.
Data exclusions	No data was excluded.
Replication	For cell types and cell lines where multiple replicates were available, these were collected and processed to assess the replicability of the selected TFs by Moni
Randomization	Not applicable
Blinding	Not applicable

Reporting for specific materials, systems and methods

We require information from authors about some types of materials, experimental systems and methods used in many studies. Here, indicate whether each material, system or method listed is relevant to your study. If you are not sure if a list item applies to your research, read the appropriate section before selecting a response.

Materials & experimental systems

n/a	Involved in the study	
☒	☐	Antibodies
☒	☐	Eukaryotic cell lines
☒	☐	Palaeontology and archaeology
☒	☐	Animals and other organisms
☒	☐	Human research participants
☒	☐	Clinical data
☒	☐	Dual use research of concern

Methods

n/a	Involved in the study	
☒	☐	ChIP-seq
☒	☐	Flow cytometry
☒	☐	MRI-based neuroimaging
