# Supplementary material for: Multiomics data integration unveils core transcriptional regulatory networks governing cell-type identity
Source: NPJ Syst Biol Appl. 2020 Aug 24;6:26. doi: 10.1038/s41540-020-00148-4 (PMC7445234; doi:10.1038/s41540-020-00148-4)
Supplement: Supplementary file 2 — Supplementary Figures [file 41540_2020_148_MOESM2_ESM.pdf]

Supplementary Figure 1

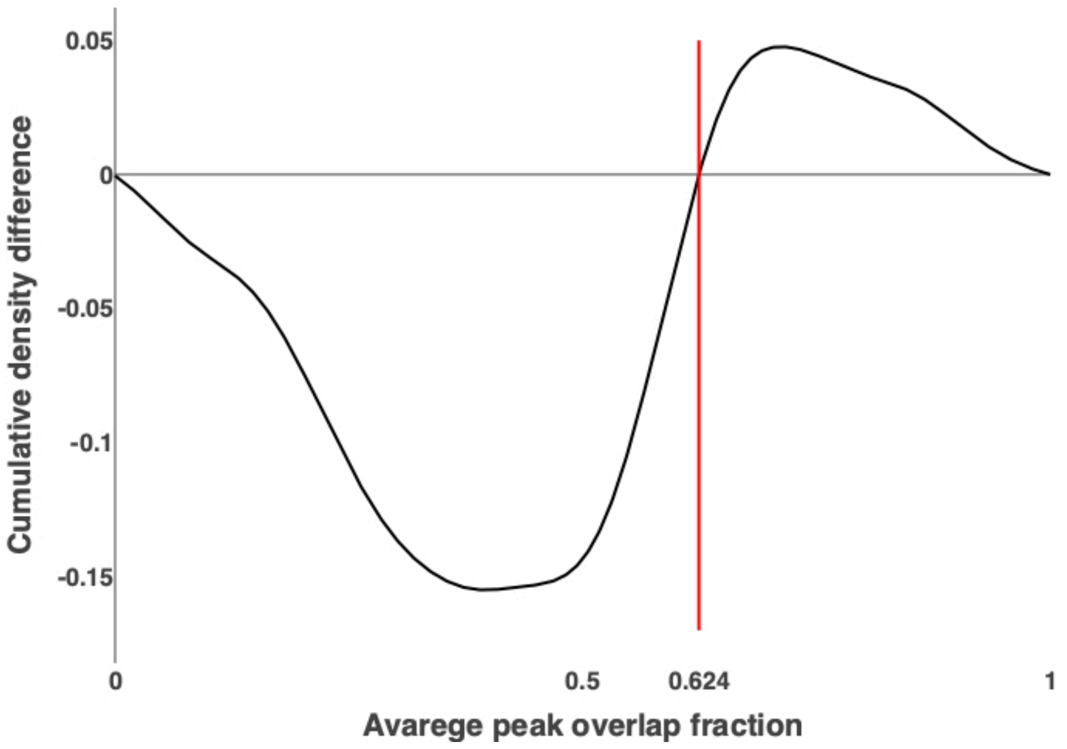

Supplementary Figure 2

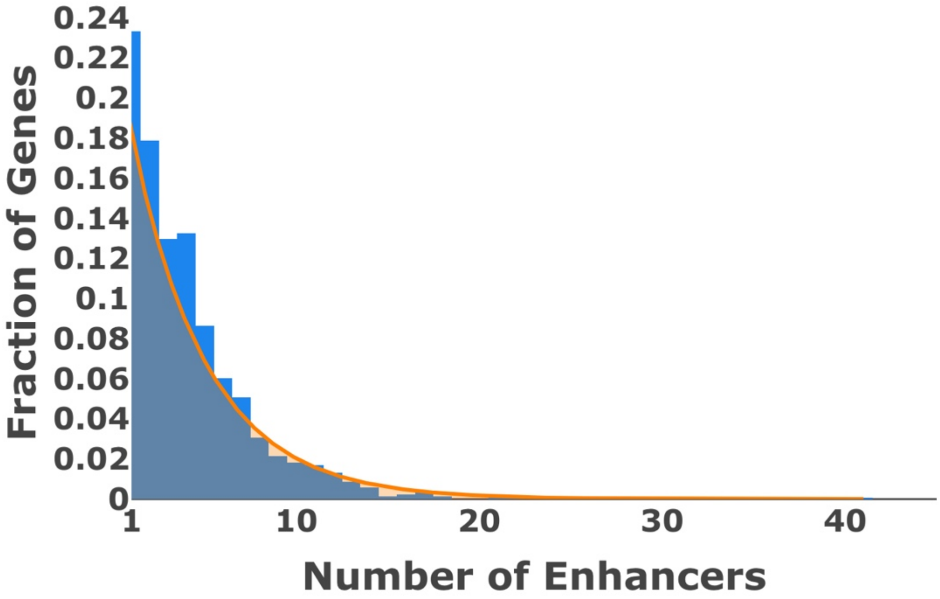

Supplementary Figure 3

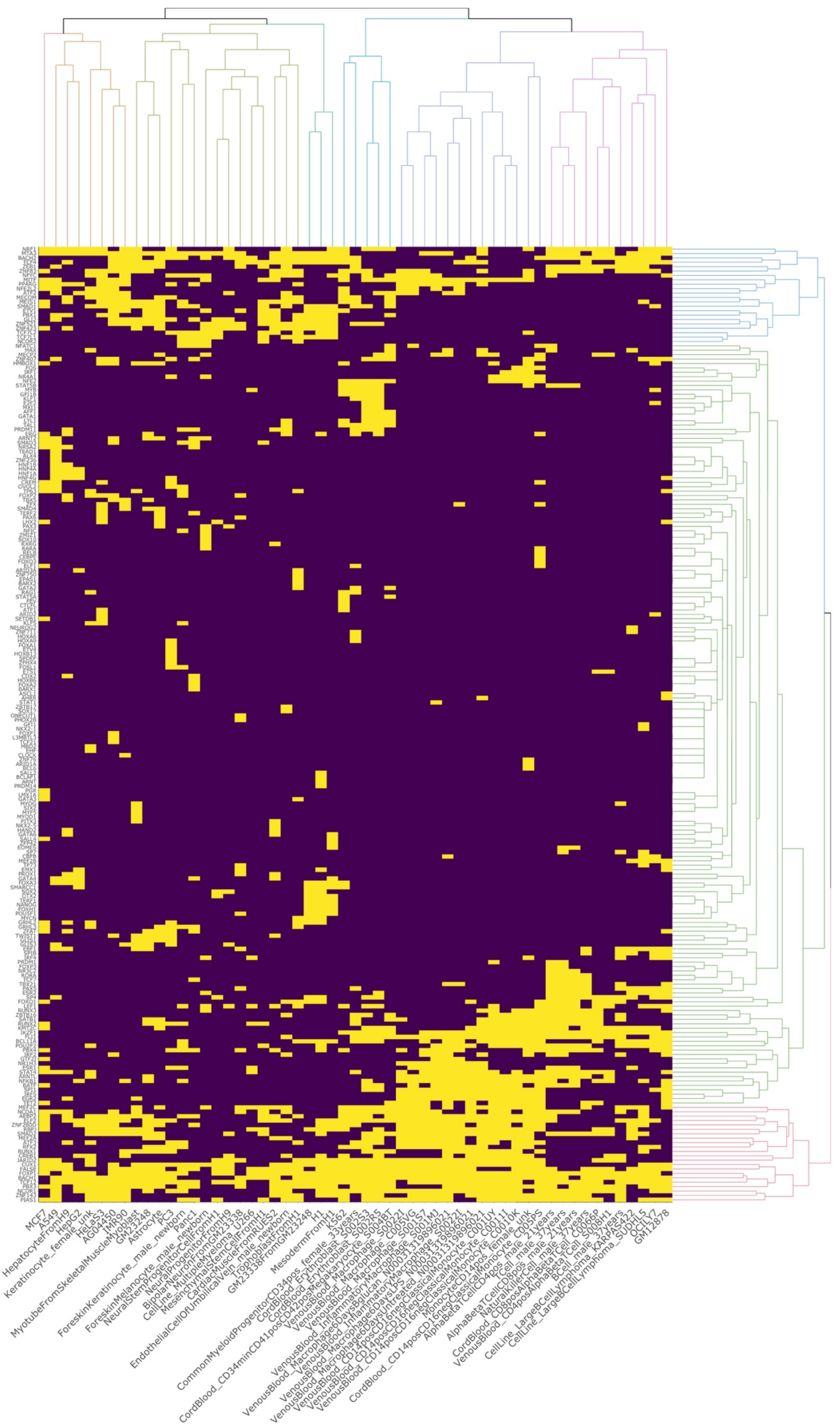
NCOA1
AEBP2
ELF2
ZNF280D
RBPJ
SMAD2
MEF2A
ATF3
RFX2
RUNX1
CREB1
JARID2
CUX1
FALSE
FOXP1
BACH1
TCF12
PBX3
NCOR1
ZNF143
PIAS1
MCF7
A549
HepatocyteFromH9
HepG2
Keratinocyte_female_unk
HeLaS3
AG04450
IMR90
MyotubeFromSkeletalMuscleMyoblast
GM23248
Astrocyte
PC3
ForeskinKeratinocyte_male_newborn
Panc1
ForeskinMelanocyte_male_newborn
NeuralStemProgenitorCellFromH1
NeuralProgenitorCellFromH9
BipolarNeuronFromGM23338
CellLine_MultipleMyeloma_U266
MesenchymalStemCellFromH1
CardiacMuscleFromRUES2
EndothelialCellOfUmbilicalVein_male_newborn
TrophoblastFromH1
GM23338FromGM23248
H1
MesodermFromH1
K562
CommonMyeloidProgenitorCD34pos_female_33years
CordBlood_Erythroblast_S002S3
CordBlood_Erythroblast_S002R5
CordBlood_CD34minCD41posCD42posMegakaryocyte_S004BT
CellLine_LargeBCellLymphoma_KARPAS422
CellLine_LargeBCellLymphoma_SUDHL5
OCILY7
GM12878

Supplementary Figure 4

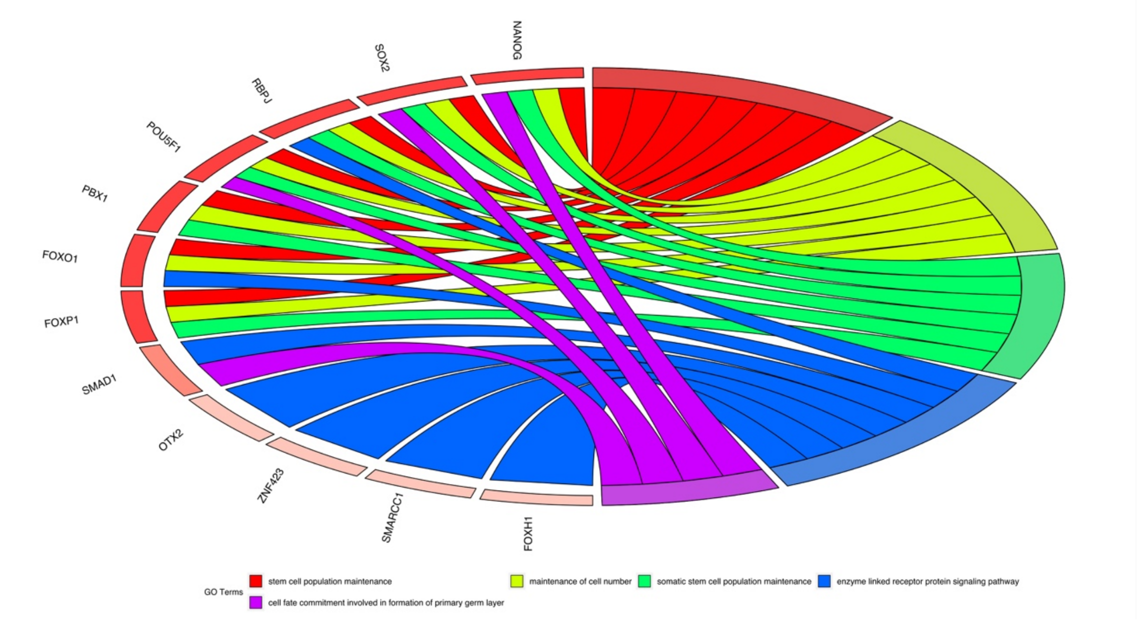

Supplementary Legends

Supplementary Figure 1: Overlap threshold for TF ChIP-seq peaks. The difference of the cumulative distribution of true positive and true negative interactions (black solid line) by the fraction of reciprocal TF binding site overlap. Above a threshold of 62.4% (red line), it is more likely to observe a true positive complex formation.

Supplementary Figure 2: Empirical distribution of enhancers per gene in 57 cell types and lines (blue) compared to the best exponential fit of the distribution (orange). The empirical distribution approximately follows an exponential distribution consistent with previous studies.

Supplementary Figure 3: Clustered heatmap of genes versus cell types/lines depicting TFs contained (yellow) in the corresponding reconstructed networks. Hierarchical clustering of TFs (rows) identifies three optimal clusters involving co-factors predominantly active in blood cells (pink), co-factors predominantly active in non-blood cells (blue) and phenotype-specific core TFs (green). Similarly, optimal hierarchical clustering of cell types/lines identifies seven

clusters: T/B cells (violet), Monocytes/Macrophages (blue), Erythroblasts/Megakaryocytes/Myeloid cells (turquois), pluripotent cells (light green), pluripotency-derived/cancer (dark green), Fibroblast/Mesenchymal cells (brown) and breast epithelial cancer (pink).

Supplementary Figure 4: Gene Ontology enrichment of iPSCs. TFs in the reconstructed core network are linked to enriched Gene Ontology terms. TFs are color-coded from white to red, where higher saturation of red indicated participation in more enriched ontology terms.
