# Supplementary material for: Multiomics data integration unveils core transcriptional regulatory networks governing cell-type identity
Source: NPJ Syst Biol Appl. 2020 Aug 24;6:26. doi: 10.1038/s41540-020-00148-4 (PMC7445234; doi:10.1038/s41540-020-00148-4)
Supplement: Supplementary file 3 — Supplementary Table legends [file 41540_2020_148_MOESM3_ESM.docx]

**Supplementary Table 1:** Gene Ontology enrichment of 57 cell types.

**Supplementary Table 2:** Validated TF complexes in H1 embryonic stem cells.

**Supplementary Table 3:** Datasets used in this study.
